# Lateral electrical transport, optical properties and photocurrent measurements in two-dimensional arrays of silicon nanocrystals embedded in SiO_2_

**DOI:** 10.1186/1556-276X-6-227

**Published:** 2011-03-16

**Authors:** Spiros Gardelis, Pavlos Manousiadis, Androula G Nassiopoulou

**Affiliations:** 1 IMEL/NCSR Demokritos, Terma Patriarchou Grigoriou, Aghia Paraskevi, 15310 Athens, Greece

## Abstract

In this study we investigate the electronic transport, the optical properties, and photocurrent in two-dimensional arrays of silicon nanocrystals (Si NCs) embedded in silicon dioxide, grown on quartz and having sizes in the range between less than 2 and 20 nm. Electronic transport is determined by the collective effect of Coulomb blockade gaps in the Si NCs. Absorption spectra show the well-known upshift of the energy bandgap with decreasing NC size. Photocurrent follows the absorption spectra confirming that it is composed of photo-generated carriers within the Si NCs. In films containing Si NCs with sizes less than 2 nm, strong quantum confinement and exciton localization are observed, resulting in light emission and absence of photocurrent. Our results show that Si NCs are useful building blocks of photovoltaic devices for use as better absorbers than bulk Si in the visible and ultraviolet spectral range. However, when strong quantum confinement effects come into play, carrier transport is significantly reduced due to strong exciton localization and Coulomb blockade effects, thus leading to limited photocurrent.

## Introduction

Silicon nanocrystals (Si NCs) embedded in dielectric matrices such as silicon dioxide or silicon nitride have unique electrical and optical properties which are determined by quantum size and Coulomb blockade effects [1-3]. A significant consequence of the quantum size effect is the bandgap opening with decreasing NC size [4,5]. This unique property of the Si NCs can be exploited in order to build absorbers for photovoltaic applications [6-12]. A fundamental problem with the existing silicon (Si) photovoltaics is that a significant part of the solar cell spectrum in the ultraviolet region, i.e., at energies much higher than the bandgap of silicon, is absorbed creating hot electrons and holes which relax to their respective band edges, losing their energy as heat through electron-phonon scattering and subsequent phonon emission. This effect poses a limit to the conversion efficiency of the cell. One way to increase the conversion efficiency beyond this limit is to use a tandem cell, i.e., a stack of absorber layers with different bandgaps to cover a larger range of the solar spectrum than a single bandgap absorber layer. These structures belong to the third generation of solar cells and are predicted to have an energy conversion efficiency limit of 60% [13].

The growth of very thin nanocrystalline (nc) Si films with thickness from 5 to 30 nm by low-pressure chemical vapor deposition (LPCVD) was reported by the authors previously [14]. Films grown by this method have columnar structures and consist of a high density of Si NCs with a very narrow size distribution and arranged in a two-dimensional (2-D) array configuration [14]. The size of the NCs in the *z*-direction is homogeneous in the whole film and equal to the film thickness, whereas in the *x*-*y *plane their size does not vary significantly with film thickness. In this work, we have grown similar nc-Si films on quartz substrates, in a range of thicknesses between 10 and 30 nm using LPCVD and subsequently oxidized them in order to form films containing Si NCs of controlled sizes embedded in a SiO_2 _matrix. The aim of this work is to use such films as absorbers in photovoltaic devices. We investigated their electrical and optical properties and measured photocurrent. We found that the electrical transport properties of the films were determined by tunneling of carriers through the SiO_2 _barriers between the Si NCs at low temperatures, whereas at higher temperatures by thermionic emission over these barriers. We also observed Coulomb blockade effects which persisted even above room temperature for the films containing the smaller Si NCs. In the case of the smaller NCs, photocurrent measurements as a function of energy showed similar dependence as that of the absorption, revealing strong absorption and photocurrent generation in visible and ultraviolet. Photoluminescence was observed only in the film which contained the smallest Si NCs (with sizes less than 2 nm), which were well isolated from each other. By comparing the photoluminescence and absorption spectra obtained from this film, we confirm the existence of an energy shift between photoluminescence (PL) and absorption, known as the Stokes shift. In addition, the PL energy is red-shifted compared with the corresponding energy predicted from the quantum confinement effect due to a pinning of the bandgap at Si NCs/SiO_2 _interfaces [15-24]. In this film, no photocurrent was observed.

## Experimental

Four films were grown on quartz by LPCVD of Si at 610°C and a pressure of 300 mTorr with thicknesses 10, 15, 20, and 30 nm [14-16,22-24]. Hereafter, we call these films A, B, C, and D, respectively. The as-grown films consisted of Si NCs touching each other and separated only by grain boundaries [14]. The films were oxidized at 900°C in order to form a silicon dioxide layer of 18 nm. This reduced the nominal thickness of the layers to 2, 7, 12, and 22 nm, respectively. It is expected that a silicon dioxide barrier layer was also formed between the Si NCs in the films during oxidation, reducing also the lateral size of the Si NCs. Current-Voltage (I-V) and photocurrent measurements were performed using the two-terminal method and an HP4140B pA Meter/DC Voltage Source. I-V characteristics were obtained at temperatures ranging from liquid nitrogen temperature up to 380 K, whereas photocurrent measurements were performed at room temperature. Aluminum (Al) electrodes for lateral conduction were defined by optical lithography and Al etching. The distance between the two electrodes was 4 μm. The Al was deposited by electron gun evaporation to a thickness of approximately 1 μm. The superficial silicon dioxide film was etched preferentially under the Al contacts by wet hydrofluoric acid chemical etching so as to form a direct contact of the metal with the Si NCs underneath. After the formation of the Al electrodes, the films were annealed in forming gas (a mixture of 5% H_2 _with 95% N_2_) at 450°C for 30 min, in order to reduce the effect of charge trapping at the Si NCs/SiO_2 _interfaces and to improve the quality of the contacts. For the photocurrent measurements, a DC bias of 5 V was applied to the electrodes, and the films were illuminated with the monochromated white light of a Xe lamp. The photocurrent spectra were normalized to the spectral irradiation intensity of the light source with the help of a Si photodiode of a known responsivity in the spectral range between 350 and 1,100 nm. Absorption measurements were performed using the same setup as that for the photocurrent measurements, by measuring the ratio of the intensity (*I*) of the transmitted light through the film, to that of the incident light (*I*_0_) as a function of the wavelength (*λ*) according to the law:(1)

where *α(λ) *is the absorption coefficient and *x *is the film thickness. Photoluminescence was excited by the 457.9-nm line of an Ar ion laser.

## Results and discussion

The as-grown films had columnar structures and consisted of Si NCs, in a 2-D array configuration, with sizes in the *z*-direction equal to the film thickness. The lateral dimension (*x*-*y *plane) of the Si NCs was in all films between 12 and 13 nm with a narrow size Gaussian distribution which did not vary much with film thickness [14]. Thermal oxidation reduced the thickness of the films and hence the vertical dimension of the Si NCs within the films. Oxidation has also reduced, to some extent, the lateral size of the Si NCs giving rise to thin SiO_2 _tunnel barriers between adjacent Si NCs.

### Electrical transport measurements

**Figure 1:**
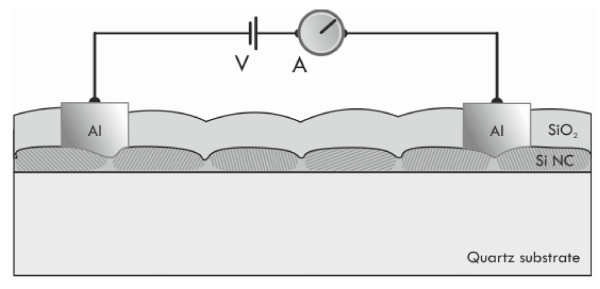
**Schematic of the film structure and of the set-up for the electrical measurements**.

**Figure 2:**
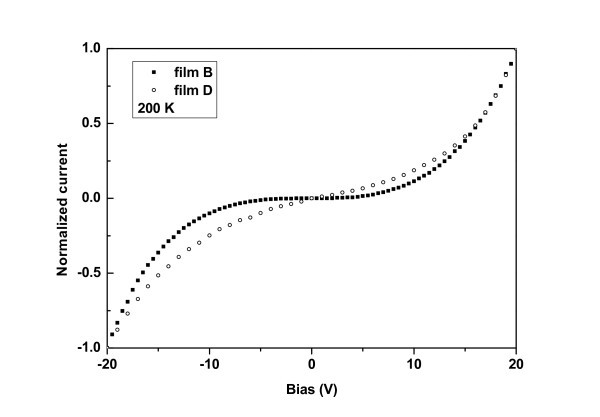
**I-V characteristics obtained from films B and D at 200 K**.

**Figure 3:**
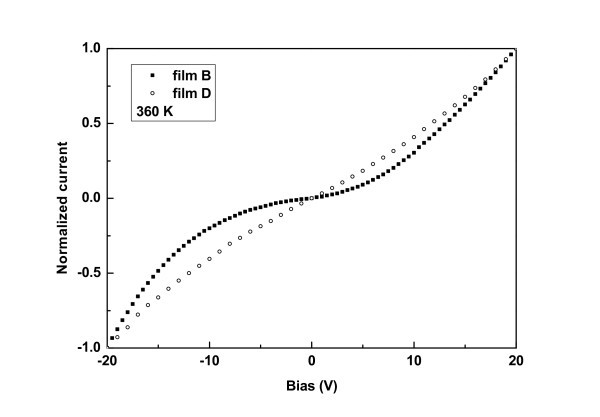
**I-V characteristics obtained from films B and D at 360 K**.

Figure 1 shows a schematic of a Si NC film and the experimental setup for the two terminal electrical measurements. Figures 2 and 3 show normalized I-V characteristics obtained from films B and D at 200 and 360 K respectively. The superlinear shape of the I-V characteristics, with a clear voltage threshold at low temperatures, was a result of the collective effect of the Si NCs involved in the transport which were separated by tunnel barriers and experienced Coulomb blockade effects due to their small sizes [3,25-29]. Indeed, it has been shown that the Coulomb blockade gap of an ordered array of identical islands and junctions exhibits a higher threshold voltage compared to a single island [30,31]. The increase of temperature caused a decrease of the threshold voltage, as carriers acquire enough energy to overcome the corresponding Coulomb blockade gaps. This was more evident in film D, which contained larger Si NCs. As a result, the I-V characteristics obtained from this film became linear at higher temperatures (see Figure 3). On the contrary, in films B and C, the I-V characteristics retained their superlinear shape even at higher temperatures. Film A was too resistive, showing an even larger threshold voltage at room temperature, whereas at lower temperatures it was impossible to measure any currents. This agrees well with the fact that the Si NCs within this film were too small and well separated with SiO_2 _potential barriers so that much larger biases were needed by the carriers to overcome the Coulomb gaps in the Si NCs.

**Figure 4:**
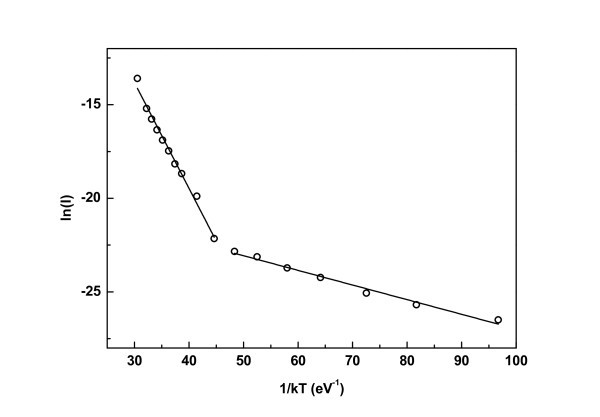
**Arrhenius plot of current, *I *for film B at a bias of 5 V**.

Temperature dependence of the current in all films revealed two main temperature regimes for transport. At low temperatures, carriers can tunnel through the SiO_2 _potential barriers between the Si NCs, whereas at higher temperatures thermionic emission is more pronounced than tunnel transport. A similar behavior has been observed, in general, in the transport of polycrystalline Si films [32-34] and of other granular semiconductors which consist of grains separated by grain boundaries [35,36]. Figure 4 shows an example of an Arrhenius plot of the current obtained from film B at a bias of 5 V as a function of *1/kT*, where *k *is the Boltzmann constant and *T *is the temperature. Activation energies *E*_1 _and *E*_2_, corresponding to thermionic emission and tunneling respectively, were extracted from these plots. For film B, *E*_1 _was calculated to be 563 meV when the applied voltage between the electrodes was 5 V. This value was reduced to 463 meV at an applied voltage of 20 V. This is expected to occur as carriers at higher applied voltages acquire higher energies, resulting in lower activation energies for thermionic emission. *E*_2 _was calculated to be 78 meV with small fluctuations around this value for different applied voltages. Similar values for *E*_1 _were extracted from the Arrhenius plots for films C and D, whereas *E*_2 _was reduced for films C to a value of 35 meV and for film D to 25 meV. We have proven elsewhere [37] that *E*_2 _is the charging energy which is needed by a carrier to overcome the Coulomb blockade gap of a nanocrystal. The larger values of *E*_2 _for film B compared to those of films C and D agree very well with the fact that larger Coulomb blockade gaps are expected for the smaller Si NCs consisting film B.

### Absorption measurements

**Figure 5:**
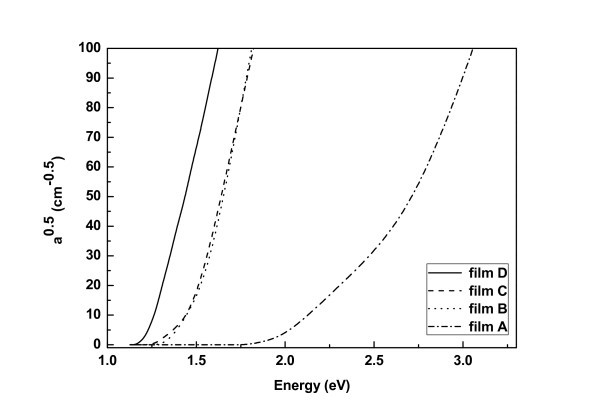
** as a function of energy of the incident light**.

Absorption measurements were obtained from all films at room temperature, as shown in Figure 5. The figure shows plots of the square root of *a*(*λ*) in Equation 1, as a function of the energy of the incident light on the film, for the different films. The linear dependence of  on the energy of illumination shows that the Si NCs within the films have indirect bandgaps. By extrapolating the linear part of the plots to  = 0, one can estimate the average bandgap *E*_g _of the Si NCs within the films. A clear bandgap upshift can be observed with decreasing nanocrystal size. For film D, *E*_g _was 1.19 eV; for film C, *E*_g _was 1.27 eV; for film B, *E*_g _was 1.32 eV. In film A, containing Si NCs with sizes less than 2 nm, we observed two linear parts in the plot  as a function of energy. These correspond to two different onsets in the absorption. The first one occurs at 1.75 eV, while the second one at 2.5 eV (found by extrapolating the second linear part of the plot to  = 0, in Figure 5). Higher absorption than in bulk Si in the visible and ultraviolet regions is observed.

### Photocurrent measurements

**Figure 6:**
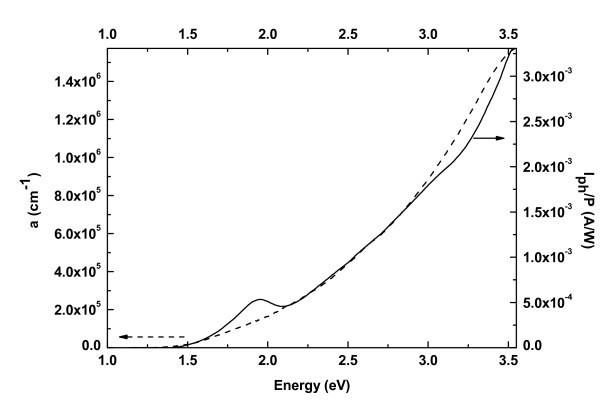
Photocurrent normalized to the power of incident light and absorption spectrum for film C.

In photocurrent measurements a DC electric field was applied between two electrodes in order to separate the photo-generated electron hole pairs and collect the electrons and holes which were generated within the Si NCs in the films by illumination with light of energy above the bandgap of the material. In Figure 6, a comparison is shown between photocurrent normalized to the power of the incident light and absorption spectra obtained from film C. The two spectra fit perfectly one upon the other, confirming that the observed photocurrent is indeed due to carriers generated in the Si NCs within the films when the energy of the illumination becomes higher that the energy bandgap of the Si NCs. The peak at 1.95 eV which is only present in the photocurrent spectra of all films is associated with a defect in the silica matrix, the so-called non-bridging oxygen hole center, which has an absorption band at this energy [38,39]. These centers can trap electrons in the silica matrix, which can be released and contribute to the photocurrent once the energy of the illumination reaches 1.95 eV.

### Electrical measurements under illumination

**Figure 7:**
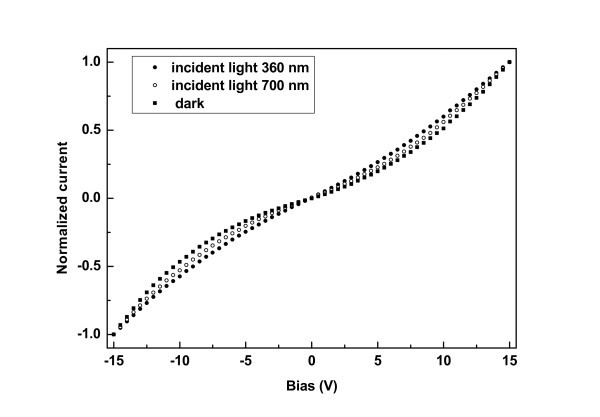
I-V characteristics for film B, in dark and under illumination.

I-V characteristics were obtained under illumination at different wavelengths at room temperature. Generally, we observed an increase of current with increasing intensity of illumination for energies above the bandgap of Si NCs within the films B, C, and D. This is expected, as photons of these energies generate electron hole pairs within the Si NCs. We also examined the role of the illumination wavelength on the photocurrent. In Figure 7, a comparison between normalized room temperature I-V characteristics obtained from film B respectively in the dark, under illumination at 700 nm (or 1.77 eV), and under illumination at 300 nm (or 3.44 eV), is shown. It is evident that the shape of the normalized I-V curves changes significantly in the case of illumination at 360 nm. The shape of the I-V curve changes from superlinear in the dark to linear under illumination at 360 nm. However, illumination at 700 nm causes a smaller change in the shape of the I-V curve. According to the above analysis of the electrical measurements, the shape of the I-V curve in the dark is determined by the Coulomb gap of the Si NCs within the film. Thus, at higher energies of illumination, carriers acquire sufficient energy to overcome the Coulomb gaps within the Si NCs, resulting in a linear I-V characteristic.

### Photoluminescence measurements

**Figure 8:**
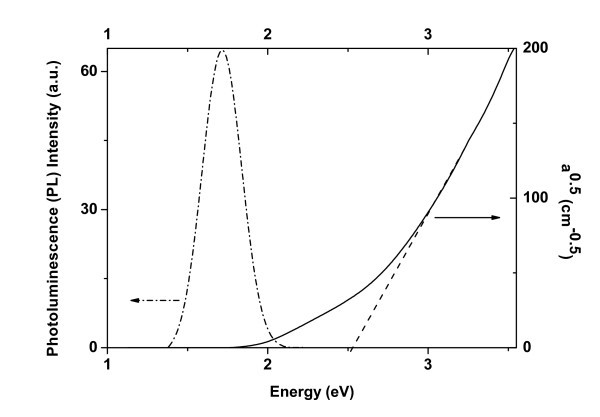
PL and absorption spectra obtained from film A at room temperature.

PL measurements were performed in all films at room temperature. Only the film A which contains Si NCs of sizes less than 2 nm, showed light emission. An example of PL spectrum is shown in Figure 8. Efficient light emission at room temperature from this film is attributed to the small size of the NCs (strong confinement) and their separation by SiO_2 _barriers. Their discrete character explains also the fact that no photocurrent was observed in this sample. By comparing the PL and absorption spectra (shown in Figure 8) we notice the following: Two different energy onsets are observed in absorption. The first occurred at 1.75 eV, whereas the second, which is sharper, occurred at 2.5 eV. The first absorption onset is within the spectral range of the PL spectrum (1.4 eV-2 eV) and is attributed to transitions involving Si NC/SiO_2 _interface states [17-24]. The second sharper absorption onset at 2.5 eV can be attributed to the energy bandgap, *E*_g_, of the Si NCs within the film. According to theoretical calculations such an energy gap corresponds to Si NCs with sizes of less than 2 nm [20,40]. This agrees well with the sizes of the Si NCs within this film.

## Conclusions

In summary, we have investigated systematically the electrical, optical, and photocurrent properties of very thin films on quartz containing Si NCs in a 2-D configuration, with sizes in the range between less than 2 and 20 nm, embedded in a silicon dioxide matrix. Strong Coulomb blockade effects in the electric transport were observed, particularly in the films containing the smaller Si NCs. Absorption measurements showed an energy upshift of the energy bandgap of the Si NCs with decreasing size. Photocurrent spectra followed absorption, revealing that photocurrent is indeed due to electron hole generation within the Si NCs. Moreover, in films containing very small Si NCs (sizes <2 nm), separated by SiO_2 _barriers, strong quantum confinement effects were observed. Excitons generated by light absorption within the Si NCs were strongly localized, and no photocurrent was measured. In these films, exciton recombination by light emission was more probable than non-radiative recombination, resulting in light emission at room temperature. This systematic study confirms that Si NCs are interesting for use as better ultraviolet absorbers than bulk Si in photovoltaic devices. However, when strong confinement comes into play in the Si NCs, one should consider strong localization effects of the photo-generated excitons that result in the absence of photocurrent.

## Competing interests

The authors declare that they have no competing interests.

## Authors' contributions

All authors contributed equally to this work. All authors read and approved the final manuscript.
